# Experimental controlled-NOT gate simulation with thermal light

**DOI:** 10.1038/srep30152

**Published:** 2016-07-21

**Authors:** Tao Peng, Vincenzo Tamma, Yanhua Shih

**Affiliations:** 1 University of Maryland Baltimore County, Department of Physics, Baltimore, Maryland 21250, USA; 2 Universität Ulm, Institut für Quantenphysik and Center for Integrated Quantum Science and Technology (IQST), Ulm, D-89069, Germany

## Abstract

We report a recent experimental simulation of a controlled-NOT gate operation based on polarization correlation measurements of thermal fields in photon-number fluctuations. The interference between pairs of correlated paths at the very heart of these experiments has the potential for the simulation of correlations between a larger number of qubits.

The discovery of the Hanbury Brown and Twiss (HBT) effect^1^,^2^ in 1956 triggered the development of the field of quantum optics. Indeed, this phenomenon motivated numerous studies of multiphoton entanglement and interference not only from a fundamental point of view^3^,^4^,^5^,^6^,^7^,^8^ but also toward applications in information processing^9^,^10^,^11^,^12^,^13^, metrology^14^,^15^ and imaging^16^,^17^,^18^.

Recent efforts have been made to simulate quantum entanglement using classical light^8^,^19^,^20^,^21^,^22^,^23^,^24^. These studies are important toward achieving a deeper understanding of the differences between classical and quantum systems. Moreover, although such schemes may suffer of an exponential scaling in the number of resources comparing with the quantum systems^25^,^26^,^27^, they make it possible to simulate small-scale quantum systems with simple interferometers without being affected by decoherence. We have recently developed a novel detection scheme that measures the photon-number fluctuation correlation(PNFC) of thermal light^28^. This scheme has been applied to the study of the multi-photon coherence of thermal states^8^,^23^,^24^,^28^,^29^,^30^, leading to effects similar to the nonlocal interference characterizing entangled states.

Motivated by these results, we experimentally demonstrate here how multiphoton interference of pairs of correlated optical paths emerges from the measurement of photon-number fluctuations of thermal fields. This phenomenon is not only interesting from a fundamental point of view but also opens the way to the simulation of quantum gate operations. In particular, by using only a pseudo-thermal source^31^,^32^ and a linear optical interferometer, a controlled-NOT (CNOT) gate operation^33^,^34^,^35^,^36^,^37^,^38^ is experimentally simulated. The experimental setup (Fig. 1) is a realization in the spatial domain^24^ of the theoretical proposal of Tamma and Seiler^8^. In particular, we demonstrate how correlation measurements in the fluctuations of the number of photons at the output of the interferometer not only simulate (Fig. 2) the truth-table of a CNOT-gate (Table 1) but also the Bell correlations (Fig. 3) typical of a CNOT-gate operation.

## Results

### Description of the experiments

**Figure 1:**
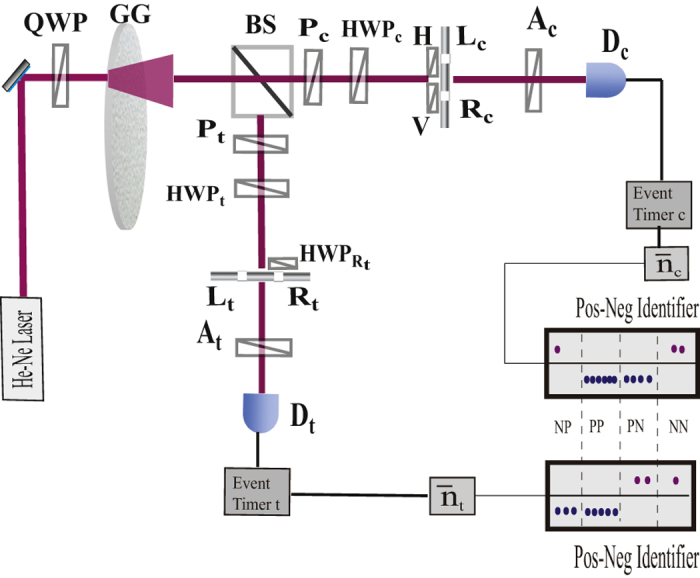
Schematic setup for the CNOT gate experimental simulation. The light emitted by a He-Ne laser is set to be left-circularly polarized. A rotating ground glass (*GG*) is then used to “thermalize” the coherent laser light into a large number of incoherent subfields. A beamsplitter (BS) splits the wavepackets into two beams. Polarizers *P*_*i*_ and half-wave plates HWP_*i*_ (*i* = *c*,*t*) are used to prepare the “control” and “target” beams at polarization angles *ϕ*_*c*_ and *ϕ*_*t*_, respectively, with respect to the horizontal direction. Each beam interacts with a mask with two pinholes *L*_*i*_ and *R*_*i*_ separated beyond the spatial coherence length of the thermal field. Two polarizers oriented in horizontal (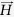) and vertical (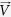) directions, respectively, are placed in front of pinholes *L*_*c*_ and *R*_*c*_. A half-wave plate 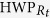, implementing a flip from *H* to *V* polarization and vice versa, is placed in front of the pinhole *R*_*t*_. *A*_*c*_–*D*_*c*_ and *A*_*t*_–*D*_*t*_ are two independent polarizer-detectors performing single-photon detections at arbitrary polarization angles *θ*_*i*_. A photon-number fluctuation correlation (PNFC) circuit is used to measure the photon-number fluctuation correlations between detectors *D*_*c*_ and *D*_*t*_.

We describe the experimental setup, depicted schematically in Fig. 1. The light source is a standard pseudo-thermal source consisting of a circularly polarized 633 nm CW laser beam and a rotating ground glass (GG). The diameter of the laser beam is ~2 *mm*. The size of the tiny diffusers on the GG is roughly a few micrometers. A large number of circularly polarized incoherent wavepackets, or subfields, are scattered from a large number of diffusers. The second-order coherence time of the source is measured to be ~90 *ms*. The randomly scattered wavepackets are then split by a non-polarizing beamsplitter into two beams, the “control beam” c and the “target beam” t. A polarizer *P*_*i*_ and a half-wave plate HWP_*i*_ prepare each beam *i* = *c*, *t* at an arbitrary polarization direction 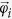 corresponding to an angle *ϕ*_*i*_ with respect to the horizontal direction. The control beam goes through a mask with two polarizers in the horizontal (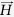) and vertical (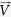) directions placed in front of the two pinholes *L*_*c*_ and *R*_*c*_, respectively. The target beam passes through two pinholes *L*_*t*_ and *R*_*t*_. A half-wave plate 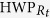, interchanging the H with the V polarization components, is placed in front of *R*_*t*_. The double-pinhole at the control arm and the double-pinhole at the target arm of the interferometer are spatially “overlapped”, i.e., *L*_*c*_ (*R*_*c*_) and *L*_*t*_ (*R*_*t*_) have equal longitudinal-transverse positions with respect to the correspondent optical axis. However, at each arm, the two pinholes are separated beyond the coherence length of the thermal field. The two light beams are then detected at the single-photon level by the two detectors *D*_*c*_ and *D*_*t*_ after passing through the polarizers *A*_*c*_ and *A*_*t*_, respectively. We consider a number *N* ~ 4 × 10^5^ of consecutive detection time intervals with width Δ*t* = 800 *μs*. The value of Δ*t* is small compared with the coherence time of the source, but large enough to guarantee enough counts per window. The registration times and the number *n*_*ij*_(*ϕ*_*i*_, *θ*_*i*_) of photodetection events at each detector *D*_*i*_ within the *j*th time window, with *j* = 1, …, *N*, are recorded for given output polarization angles *θ*_*i*_ by two independent but synchronized event timers. At each detector *D*_*i*_ the mean photon number 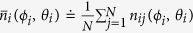 is obtained by averaging over all the values of photon number *n*_*ij*_(*ϕ*_*i*_, *θ*_*i*_) recorded in each of the N time windows *j*. The photon number fluctuation for each time window is calculated as^28^


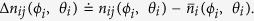


Finally, for given input polarization angles *ϕ*_*c*_ and *ϕ*_*t*_ of the control and target beams, respectively, the correlation


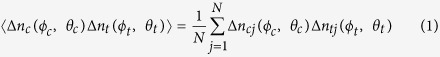


in the photon-number fluctuations is measured at the output for arbitrary polarization angles *θ*_*c*_, and *θ*_*t*_.

### Interference between pairs of correlated paths and CNOT-gate simulation

**Figure 2:**
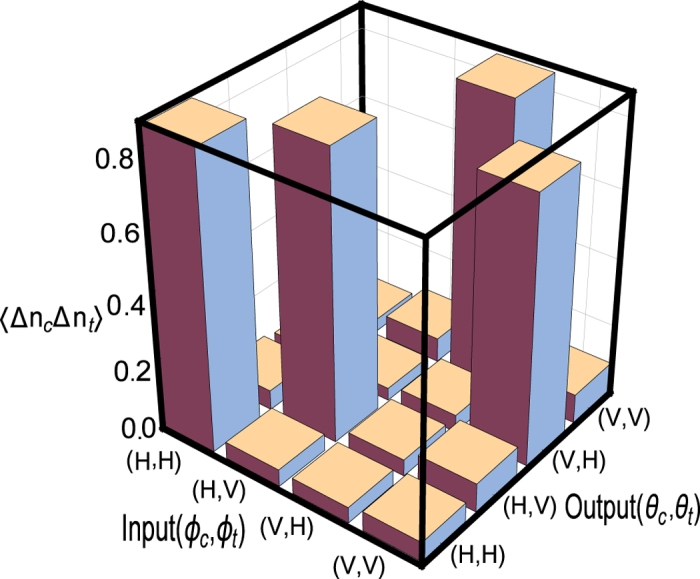
Experimental observation of the polarization correlation 〈Δ*n*_*c*_(*ϕ*_*c*_, *θ*_*c*_)Δ*n*_*t*_(*ϕ*_*t*_, *θ*_*t*_)〉 in the photon-number fluctuations for the input polarizations (*ϕ*_*c*_, *ϕ*_*t*_) = (*H*, *H*), (*H*, *V*), (*V*, *H*), (*V*, *V*) and the output polarizations (*θ*_*c*_, *θ*_*t*_) = (*H*, *H*), (*H*, *V*), (*V*, *H*), (*V*, *V*). For each input polarization (*ϕ*_*c*_, *ϕ*_*t*_), the plotted data are normalized by 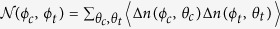.

**Figure 3:**
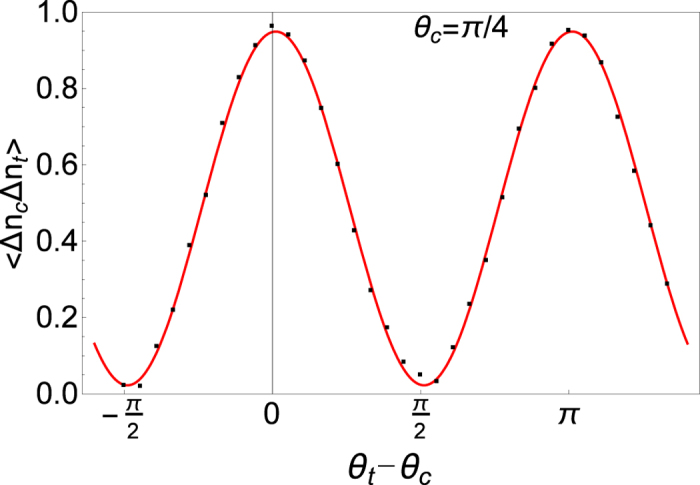
Experimental observation of the polarization correlation 〈Δ*n*_*c*_(*ϕ*_*c*_, *θ*_*c*_)Δ*n*_*t*_(*ϕ*_*t*_, *θ*_*t*_)〉 in the photon-number fluctuations for the input polarizations *ϕ*_*c*_ = *π*/4 and *ϕ*_*t*_ = 0. The black dots are experimental data normalized by 〈*n*_*c*_(*ϕ*_*c*_, *θ*_*c*_)〉〈*n*_*t*_(*ϕ*_*t*_, *θ*_*t*_)〉, and the continuous red sinusoidal curve is a theoretical fitting based on Eq. (2). In this measurement, *θ*_*c*_ was fixed at *π*/4 and the values of *θ*_*t*_ range from −*π*/4 to 7*π*/4.

We consider first the case of input and output polarizations either in the horizontal direction 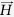 or in the vertical directions 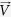. In this case, the experimental outcomes in Fig. 2 for the correlation in the photon number fluctuations in Eq. (1) simulate the truth table (Table 1) of a CNOT-gate. The initial polarization direction 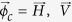 of the control beam remains always unchanged at the output. In particular, if the control beam is H-polarized then it can pass only through the pinhole *L*_*c*_ and a non-zero correlation in Eq. (1) is measured only when the target beam passes through the pinhole *L*_*t*_ without changing its initial polarization. On the other hand, a V-polarized control beam can only propagate through the pinhole *R*_*c*_ and a nontrivial correlation at the output occurs only if the target beam, by taking the path *R*_*t*_, flips its polarization direction from 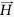 to 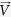 or vice versa. These experimental results witness the emergence of two pairs of correlated paths corresponding to the propagation through either the pinhole pair (*L*_*c*_, *L*_*t*_) or the pair (*R*_*c*_, *R*_*t*_). Can these pairs of correlated paths actually interfere? One may think that this is not possible since the two pinhole pairs are placed with respect to each other beyond the source coherence length. Interestingly, we show here experimentally that interference not only occurs but allows also us to fully simulate the entanglement operation of a CNOT gate. For this purpose, we consider the case where the control beam is polarized at an angle *ϕ*_*c*_ = *π*/4 corresponding to the direction 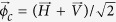. In this case, by considering a target beam in the initial polarization direction 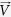, the correlation of the photon-number fluctuations in Fig. 3 measured at the interferometer output is given by


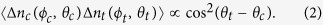


Indeed, the measurement simulates with ~100% visibility the polarization correlations typical of the Bell state 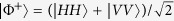 produced at the output of a “genuine” CNOT gate with input state |*ϕ*_*c*_〉|*ϕ*_*t*_〉, where 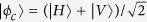 and |*ϕ*_*t*_〉 = |*V*〉. Interestingly, entanglement correlations analogous to a CNOT operation are simulated here by using only a separable input state and taking advantage of the interference between two pairs (*L*_*c*_, *L*_*t*_) and (*R*_*c*_, *R*_*t*_) of correlated paths, as will become more evident in the theoretical description in the next section.

### Theoretical description

Here we provide a theoretical analysis based on the Glauber-Scully theory^39^,^40^ of the experimental results described in the previous section. We start from modeling the state of the pseudo-thermal field. The ground glass contains a large number of tiny randomly shaped scattering diffusers, roughly a few micrometers in size. A large number of subfields or wave packets are scattered from the laser beam with random phases by these tiny diffusers. We consider each scattering diffuser as a sub-source. By considering, for simplicity, monochromatic light, the state of the pseudo-thermal field can be expressed in the coherent state representation as^41^


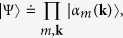


where **k** is the transverse wavevector. |*α*_*m*_(**k**)〉 is an eigenstate of the annihilation operator 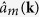 with an eigenvalue *α*_*m*_(**k**) which contains a real-positive amplitude *a*_*m*_(**k**) and a random phase *φ*_*m*_(**k**) arising from the scattering process associated with the *m*th diffuser.

We can then evaluate, for given input polarization angles *ϕ*_*c*_ and *ϕ*_*t*_, the photon-number correlation


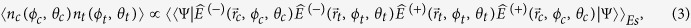


where 〈…〉_*Es*_ denotes the ensemble average over all the possible values of *α*_*m*_(**k**). Here, the field operator can be expressed as the sum


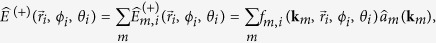


with *i* = *c*, *t*, where 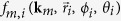 is an effective spatial transfer function (to be defined later) which takes into account the polarization dependent evolution from the *m*th pointlike diffuser to the pointlike detector *D*_*i*_ at position 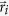.

By introducing the “effective wavefunction”


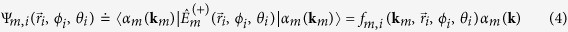


Eq. (3) becomes


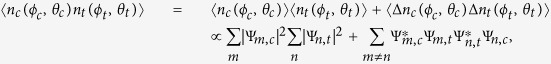


leading to the correlation between the photon-number fluctuations:


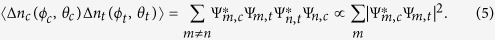


Here, the approximation in the second step of Eq. (5), given the large number of subfields, is used to simplify the notation.

We explicitly address the propagation through the two pinholes *L*_*i*_ and *R*_*i*_ at positions 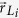 and 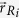, respectively, at each interferometric arm in Fig. 1 by rewriting Eq. (4) as


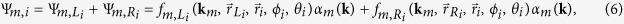


with


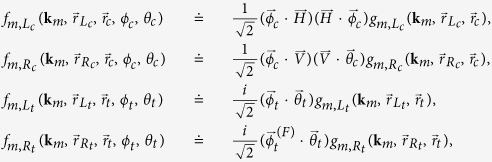


where 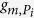 is the Green’s function associated with the spatial propagation from the *m*th subfield to the detector *D*_*i*_ passing through the pinhole *P*_*i*_ (*P* = *L*, *R*), and “F” indicates the flip in the polarization components (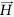 to 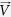 and vice versa) of the polarization direction 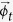 performed by the waveplate 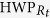.

By substituting Eq. (6) in Eq. (5) we obtain


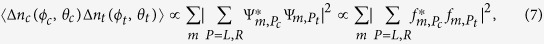


where, in the second step of Eq. (7), the value |*α*_*m*_(**k**)|^2^ was assumed to be the same for each subfield *m*.

Since the pinholes *L*_*i*_ and *R*_*i*_ are placed with respect to each other beyond the transverse coherence length of the thermal field, Eq. (7) reduces to^24^


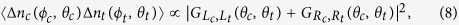


with


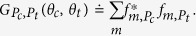


Interestingly, the measured correlation in the photon-number fluctuations emerges from the interference between only two multiphoton contributions 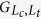 and 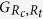 associated with the propagation through the two pairs of pinholes (*L*_*c*_, *L*_*t*_) and (*R*_*c*_, *R*_*t*_), respectively.

We recall now that in the experiment, the two detectors are placed along the optical axes in the control and target arms of the interferometer and the two pinholes in each arm are at the same distances from the axes. In these conditions Eq. (8) becomes^24^


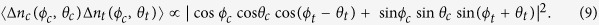


We now compare this result with a genuine CNOT entangling operation on the input state |*ϕ*_*c*_〉|*ϕ*_*t*_〉, where 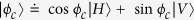 and 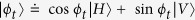, leading to the output entangled state


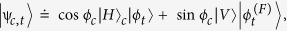


where 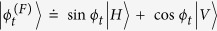. Polarization correlation measurements over the state |ψ_*c*,*t*_〉 occur with a probability


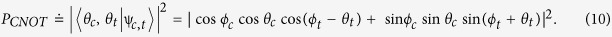


Comparing Eq. (10) with Eq. (9), it is clear that the measurement of correlations between the photon-number fluctuations at the two output ports leads to the simulation of a CNOT gate operation.

## Discussion

In summary, we have experimentally demonstrated for the first time thermal light interference between two pairs of correlated paths, where each path in a pair is spatially incoherent with the paths in the other pair. This counterintuitive effect is at the very heart of the experimental simulation of a CNOT gate operation described here.

In particular, the simulation of the entanglement correlations typical of a CNOT-gate operation emerges from the interference between the two pairs of paths (*L*_*c*_, *L*_*t*_) and (*R*_*c*_, *R*_*t*_) in Fig. 1 propagating through two corresponding pairs of pinholes when correlation measurements in the photon-number fluctuations are performed at the output. Interestingly, this interference phenomenon occurs even if the pinholes in one pair are separated by more than the source coherence length with respect to the pinholes in the other pair.

Furthermore, the correlation in the photon-number fluctuations between the polarizations measured by the two distant detectors resembles the typical nonlocal behavior of entangled states even if no entanglement process occurs in the interferometer. Indeed, by not relying on complex non classical interferometers, the interference operation demonstrated here is apparently insensitive to photon losses and decoherence.

Lastly, by taking advantage of the abundant source of input states characterizing a thermal source with respect to single photon sources, this phenomenon can be used, in principle, to simulate correlations between a larger number of qubits, with potential applications in novel optical algorithms^8^,^42^,^43^,^44^,^45^,^46^, imaging and metrology^8^,^18^,^24^.

## Additional Information

**How to cite this article**: Peng, T. *et al*. Experimental controlled-NOT gate simulation with thermal light. *Sci. Rep.* **6**, 30152; doi: 10.1038/srep30152 (2016).

## Figures and Tables

**Table 1:** Truth table for a CNOT gate operation.

Input state	Output state			
	**HH**	**HV**	**VH**	**VV**
HH	1	0	0	0
HV	0	1	0	0
VH	0	0	0	1
VV	0	0	1	0
